# Exposing the key functions of a complex intervention for shared care in mental health: case study of a process evaluation

**DOI:** 10.1186/1472-6963-8-274

**Published:** 2008-12-23

**Authors:** Richard Byng, Ian Norman, Sally Redfern, Roger Jones

**Affiliations:** 1 Primary Care Group, Institute of Health Services Research, Peninsula Medical School, University of Plymouth, Plymouth UK; 2 School of Nursing, King's College London, UK; 3 National Nursing Research Unit, King's College London, London, UK; 4 Department of Primary Care and General Practice, King's College London, London, UK

## Abstract

**Background:**

Complex interventions have components which can vary in different contexts. Using the Realistic Evaluation framework, this study investigates how a complex health services intervention led to developments in shared care for people with long-term mental illness.

**Methods:**

A retrospective qualitative interview study was carried out alongside a randomised controlled trial. The multi-faceted intervention supported by facilitators aimed to develop systems for shared care. The study was set in London. Participants included 46 practitioners and managers from 12 participating primary health care teams and their associated community mental health teams. Interviews focussed on how and why out comes were achieved, and were analysed using a framework incorporating context and intervening mechanisms.

**Results:**

Thirty-one interviews were completed to create 12 case studies. The enquiry highlighted the importance of the *catalysing, doing and reviewing *functions of the facilitation process. Other facets of the intervention were less dominant.

The intervention *catalysed *the allocation of link workers and liaison arrangements in nearly all practices. Case discussions between link workers and GPs improved individual care as well as helping link workers become part of the primary care team; but sustained integration into the team depended both on flexibility and experience of the link worker, and upon selection of relevant patients for the case discussions. The *doing *function of facilitators included advice and, at times, manpower, to help introduce successful systems for reviewing care, however time spent developing IT systems was rarely productive. The *reviewing *function of the intervention was weak and sometimes failed to solve problems in the development of liaison or recall.

**Conclusion:**

Case discussions and improved liaison at times of crisis, rather than for proactive recall, were the key functions of shared care contributing to the success of Mental Health Link. This multifaceted intervention had most impact through *catalysing *and *doing*, whereas the *reviewing *function of the facilitation was weak, and other components were seen as less important.

Realistic Evaluation provided a useful theoretical framework for this process evaluation, by allowing a specific focus on context. Although complex interventions might appear 'out of control', due to their varied manifestation in different situations, context sensitive process evaluations can help identify the intervention's key functions.

## Background

Internationally there is a strong emphasis on 'service redesign' as a means of achieving both better quality and value for money. In the UK, providers and commissioners work together to design alternative ways of delivering services, often with the aim of improved access and care closer to home. The services themselves will often involve more collaborative and patient centred care, requiring the development of new systems, roles and skills. Furthermore assertive implementation strategies are required for overcoming resistance to change. Measuring the effectiveness of redesign requires stand alone but complex interventions to be developed and submitted for evaluation.

Whether these interventions, designed to promote shared care and other system redesign, fail or succeed will depend on both the conceptual basis of the intervention and the implementation. Evaluating complex interventions involves determining both *whether *and *how *they work; such evaluations are theoretically and practically complex. First, the components of care themselves may interact positively or negatively with each other [1] and also may be required in different doses or formats depending on context [2]. The kind of interventions required to bring about change are also likely to be multi-faceted; educational, audit-based and facilitation-based interventions each have evidence to support them [3]. The choice and application of appropriate outcome measures at different levels of change is challenging [4]. Lastly, implementation often occurs within the shifting sands of health services reform and development [2,5].

Debates about the best means of evaluating complex interventions have been particularly contested in the literature on North American social programmes where quantitative evaluations had failed to demonstrate consistent effectiveness [6]. Many researchers began to favour detailed qualitative methods over quasi experimental studies which, while potentially useful for local interpretation, were not seen as generalisable to other contexts [7]. Replications of successful 'models' in other settings often failed, leading to a loss of faith in both complex social interventions and in the ability of evaluation methodologies to pinpoint critical ingredients [8].

More recently the MRC has recognised the need for multi-faceted approaches to change in health care and outlined a staged approach for cluster randomised controlled trials (RCTs) of complex interventions, allowing for heterogeneity of the intervention in early stages, but demanding a specified model in the fully powered RCT [4,9]. The primacy of the RCT is therefore maintained, and although theoretical modelling is recommended, deriving explanations about how an intervention has its effect are not prioritised. Process evaluations of health care interventions have also broken down complex interventions into component parts and monitored the acceptability, implementation and impact of each part [10,11]. However, interactions between the parts, as well as with the surrounding context, suggest that to process evaluation will not lead to an understanding about the system as a whole [2,11]. Evaluations which prioritise an understanding of the interaction with context are rarely used in current health services research and poorly developed elsewhere [2,10]. An exception is the Southampton Heart Integrated Care Project (SHIP) which used qualitative methods to understand the negative results of an RCT and demonstrated how different recipients made sense of the intervention [12]. Hawe et al. argue that in order to be context sensitive, interventions should not be fully standardised yet can still be subjected to randomised controlled trials [5]. They suggest that the intervention needs to be evaluated and understood in terms of its 'essential' or key functions, as well as the form of the components as manifested in different contexts. There is an analogy with the concept of genotype and the resulting phenotype, which depends on an interaction with the environment.

Realistic Evaluation is a relatively new framework for understanding how and why interventions work in the real world [8] and has been recommended as a means to understand the dissemination of service innovation [13]. Analysis focuses on uncovering key mechanisms and on the interactions between mechanism and context in order to develop 'middle range theories' [14] about how they lead to outcomes. Accumulation of evidence, which may be qualitative or quantitative, and may also be derived from external sources, leads to a refining of these theories. Realistic Evaluation, therefore, promises to be a useful framework for understanding the key functions of an intervention by examining its relationship with the context.

### Shared care for long-term mental health problems

Shared care [15] and chronic disease management, [16]as complex strategies for change, have much in common and have received increasing attention over the last twenty years. However the means by which they exert an effect are poorly understood. Shared care emphasises the need for co-ordination between primary care and specialists to reduce duplication and address unmet need; chronic disease management focuses on service redesign in primary care incorporating timely review, expert input, patient involvement and information systems. These principles underpinned the development of the Mental Health Link intervention as a means of improving shared care of patients with long-term mental illness (LTMI) [17]. The principles continue to be of significant importance for health care systems undergoing whole scale system redesign [18]. Like other complex interventions, programmes of shared care have been evaluated using qualitative and quantitative methods. There is a need to develop more refined methods of evaluation that inform us about both the effectiveness and the critical components of health service interventions working at interfaces within the broad health and social care system.

### The Mental Health Link intervention and RCT

**Figure 1:**
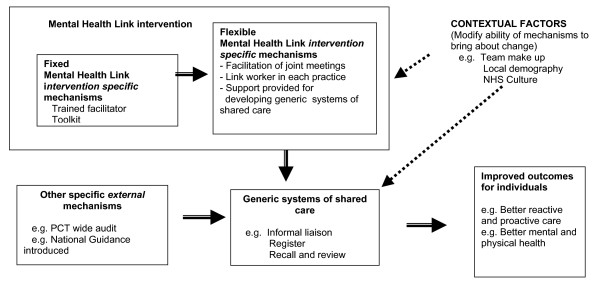
**Theoretical Model: Linking intervention specific, external and generic mechanisms to improved health care**.

The Mental Health Link intervention was designed to improve the care of patients with long-term mental illness (LTMI), looked after by family doctors (general practitioners) working in primary health care teams (PHCTs) and community mental health workers working in community mental health teams (CMHTs). The multi-faceted Mental Health Link intervention was subjected to a cluster RCT, with randomisation by practice [19]. The proposed components of shared care included: primary care-based systems for registers, recall, and review; education and audit; and the development of a liaison relationship with specialists; these generic components, the systems of shared care, were derived from the literature [17]. Delivery of organisational change was dependent on three fixed components: training of facilitators, a toolkit and small financial incentives. The toolkit included: a guide through a series of meetings attended by representatives of both teams and service users; instructions for creating registers, carrying out audits and assessing educational needs; and a flexible template for a written shared care agreement between providers, detailing allocation of responsibilities and protocols for formal communication. In contrast, the actual work of the facilitator was designed to be *explicitly flexible*, responding to the context of primary care, specialist teams and health needs, but encouraging both teams to develop shared care in line with the proposed model. Similarly the role of the linked specialist worker for each practice would depend on local context. The only generalisable *fixed *components of the intervention were, therefore, the training received by the facilitators, the payments and the toolkit. The relationship between these fixed and flexible components of the intervention, the external context, which can modify their effects and the anticipated health outcomes is depicted as an *a priori *theoretical model in Figure 1.

The RCT results revealed reduced relapse rates and improved practitioner satisfaction [19]. However, no improvements were documented in the records for physical and mental health care, and patient unmet need and satisfaction did not improve. Statistically significant differences in systems for review and liaison arrangements were found when comparing control and intervention practices. However, although link workers were allocated in all intervention practices, and registers developed in most, many intervention practices reported not developing key components of shared care (such as systems for proactive recall), and link worker activity was not always sustained. This case study examines the use of Realistic Evaluation as a framework for a context sensitive process evaluation, accompanying an RCT, designed to unpick the complexity of the intervention by examining interactions between components and context and then further defining its core functions. Our previous paper primarily describing the methodology, provided examples of how the case studies and cross case analysis were carried out, and a detailed critique of the method and the use of Realistic Evaluation [20]. This paper builds a picture of *how *the intervention, as a whole, had its effects and how the process evaluation adds meaning to the results of the trial. It then builds on existing theoretical models for evaluating complex interventions to suggest how key functions relate to external context [4,5].

## Methods

This Realistic Evaluation was based on retrospective interviews with practitioners and managers involved in developing services within both intervention and control arms of the trial. The interview process and subsequent analysis used the experience of these stakeholders as a lens to reveal and define the key functions of the intervention, and to understand the interactions between the pre-planned components and the local context.

A total of 31 individual and group interviews were completed to create 12 case studies. Sampling was purposeful: nine PHCT-CMHT pairs from the intervention practices, with a range of reported improvements, and three, which had achieved significant service improvements, from the control arm. Respondents included 21 GPs, 8 community mental health workers, 7 practice managers, 4 mental health managers, 3 practice nurses, 2 psychiatrists, a practice counsellor and a facilitator. The participants were sent information about the aims of the study prior to interviews, which were tape-recorded. Each interviewee was asked whether key health and service development outcomes of importance had occurred. Interviews then focused on developing a shared understanding about why these outcomes had or had not been achieved. The interviewer explicitly asked about the role of contextual factors and how these had an impact on the functioning of the intervention and the development of processes of shared care [8].

The interviews were transcribed and coded, based on the Realistic Evaluation framework: identifying specific contexts within and outside the practice; assigning pre-planned and emergent components of the MHL intervention and other significant external occurrences (such as a PCT audit) as mechanisms; and developing a framework for coding a range of expected and unexpected outcomes, including shared care developments and improved health care. This coding scheme relates to the theoretical basis of the intervention depicted in figure 1. Interviews with respondents from each PHCT-CMHT pair were used to construct 12 individual case studies representing the changes to relationships, systems and outcomes which had occurred during the period of the trial for each PHCT-CMHT pair studied. These case studies incorporated the context-mechanism-outcome (CMO) configurations used in Realistic Evaluation studies. Themes emerging from the detailed coding and construction of case studies were noted.

The cross case analysis utilised a modified form of analytic induction [20,21] to examine the empirical data of the case studies, and iteratively build 'middle range theories' [14] designed to be of interest to policy makers and practitioners elsewhere. Coded data were reduced to 'predictor-outcome matrices' each encompassing all 12 case studies and incorporating the CMO configurations [22]. These matrices were used to facilitate a systematic approach to develop and adapt theory. Broad outcomes of shared care, such as 'integration of link workers into primary care teams' and 'proactive review of care', were the focus for each matrix, which were then used for each cycle of analytic induction. 'Positive' cases were those cases where the outcome of interest had been achieved, at least in part, in that practice; 'negative cases' were where it had not. Potentially interacting predictors included contexts internal and external to the practices and mechanisms, both part of the intervention (eg elements of facilitation) and external influences.

Provisional causal hypotheses, ready to be tested using the analytic induction process, were derived both from themes emerging during the initial coding process, and by examining the matrix for obvious patterns. Each provisional hypothesis was further developed by systematically assessing it against first the positive and then the negative cases in the matrix, and adapting it by incorporating further contextual factors and mechanisms to describe how each overall outcome of shared care developed. Alternatively they were rejected if the data did not support the provisional hypothesis. These more refined theories about how and why services had developed, incorporating the interaction between context and mechanisms, were then rechecked against the original transcripts and further refined if necessary. Lastly, the refined hypotheses were examined together to look for overarching themes explaining the key functions of the intervention, both those relating to shared care for mental health, and those relating to successfully achieving change.

The final stage of the analysis for this paper involved setting the results of the Realistic Evaluation alongside those of the randomised controlled trial and drawing inferences about the reasons for the mixed results of the randomised controlled trial. Ethical approval was obtained for both the RCT and the Realistic Evaluation.

## Results

The extent of development of the elements of shared care and perceived success in the twelve practices varied considerably, as was to be predicted from the selection process. This reflected the variance of self reported change in shared care reported in the trial, [19] although the interviews enabled a more in-depth understanding of what constituted progress in the eyes of the practitioners. In addition, the practitioners defined some additional elements of shared care, such as 'integration of link worker into PHCT'. Table 1 shows a range of the outcomes investigated, and component indicators derived from the interviews.

**Table 1:** Outcomes and component indicators investigated in interviews

Placement or allocation of a link worker for the practice
Allocation prior to trial
Allocation prior to first meeting with practice
Allocation following initial meetings
Integration of the link worker into the team
Joint work on systems
Development of trust in link workers' activity
Discussion of clinical cases
- in meetings
- one to one
- reviewed from register
- reactively as problems arose
Development of a disease register for LTMI
Paper based register
Register based on coded diagnoses on practice computer system
Development of a database of key information about each patient with LTMI
Use of externally developed data set
In house development of clinical IT system to collect data
Use of paper based recording sheet for key data
Improvements in physical health care provision
Recall of patients for physical health checks
Improvements in proactive mental health care provision
For patients with psychosis
For patients with other long-term conditions
Improvements in reactive mental health care provision
For those under specialist care
For those under primary care only

The cross case analysis, used analytic induction cycles to develop and then refine the 'middle range theories' into statements. Examples of those statements which added to our understanding of how the intervention worked are included in Table 2. These statements cast light on both the change management functions of the intervention, as well as shared care of those with LTMI more generally. The statements provide answers to the question: 'what works for whom, in what circumstance?'

**Table 2:** Middle range theories derived from cross case analysis

**Liaison function**

*Discussions about individual patient cases both results in better care and forms the basis for developing an ongoing liaison relationship. The nature of the forum for the case discussion varies (telephone, routine group meeting, etc) and is context dependent.*

*In the absence of liaison being a policy requirement, sustained joint work requires a receptive context in both teams.*

*Initial analysis of the situation to agree an appropriate, context-dependent, form of liaison and subsequent review of progress are essential ingredients for successful liaison.*

**Developing systems**
*Facilitators can only act as catalysts for developing systems in motivated and stable practices*

*Financial support, guidance and hands on support for developing systems from facilitators are important to practices with less IT expertise and poor systems in other areas of chronic disease management*

*Efforts invested by practices and facilitators to develop 'in house' IT systems for capturing mental health data and prompting best practice do not improve chronic disease management if the new systems are cumbersome or not integrated with systems for proactive review*

*Review of progress by external facilitators contributes to solving implementation problems*

**Improved mental health care**
*Improvements in care at times of crisis follow liaison case discussions: these rely on the efficient identification of those to be discussed and a forum for the discussion.*

*In the presence of an engaged linked specialist worker and a 'working register', a regular, appropriately attended, organised forum with appropriately identified cases for shared review, can result in improved identification of unmet mental health care needs and improved care.*

### Developing shared care

A positive feedback cycle occurred when link workers became integrated into primary care teams, with productive case discussions also leading to increased trust and generating further liaison opportunities [20]. These productive discussions about individual clinical cases manifested themselves

in different ways in different contexts, and performed a key function of the intervention, in terms of the liaison process. Practitioner attributes were seen as critical to the benefit of liaison: '*there's her experience, ..... She's senior enough to .... to be able to make decisions and ..........partly of course she has a very calming influence, because that's how she is.'*

Productive clinical case discussions were reported to be the key to both link worker integration and better outcomes. These were assisted by establishing routine times for joint discussion of clinical cases; these might be at set times in practice meetings, specific meetings for mental health or during day to day contact between link worker and GP. This improved liaison was reported to be effective at promoting shared care when patients were in crisis, whether or not they were under specialist supervision. On the other hand, where productive clinical case discussion came to an end, or had never really taken off, the image of whole link working project was tarnished; one practitioner reported *'there's no point having a link worker coming to practices discussing cases which barely change from time to time.' *Productive link working was also vulnerable to changes in personnel and crises within teams. This contrasted significantly with the two large practices (one control and one intervention) which had practice based community psychiatric nurses, who had been in post for years and, as well as liaison, provided an efficient system of practice based care jointly with GPs for relatively large caseloads of patients with LTMI.

On the other hand, the negative cases in the cross case analysis demonstrated the lack of progress made by teams in developing proactive care. While registers were established in most practices, in only one practice was a link worker involved in helping review patients' records; and no practices set up systems for proactively reviewing mental health care by inviting patients in. Three well organised practices had spent significant time, with assistance from facilitators, developing 'in house' IT systems for recording patients' clinical data. Frustrations with these ventures had resulted in pessimism about the mental health link project despite initial good intentions: *'it felt like we had failed. And when it had failed... it felt we were kind of useless somehow.' *Mental Health Link had become a 'heartsink' project.

Furthermore the external environment was not a sufficiently supportive context for the scope of the proposed shared care developments: it was seen as *'a big project'*. The mental health trust management would always prioritise ensuring that each community mental health worker managed their 'case load' of people with severe mental illness, with link working seen as a lesser priority. General practices, as small businesses, whose core business is the reactive care for large populations, had few incentives to engage in thinking about these difficult issues. These issues highlighted a clear priority for shared care: setting up simple systems for proactive reviews should be favoured over the development of in-house templates and databases.

### MHL as a change management agent

The interviews revealed an ambivalence about and in some cases a lack of awareness of the toolkit which had been given to each team. The relatively modest financial rewards were seen as a token with the exception of a couple of small practices who bought in extra nursing or administrative time. In contrast, the facilitators' actions were considered important; while most comments were positive, some key omissions and several problematic interventions were mentioned. The key change management functions of the facilitation, as an adjunct to 'service development as normal', were *catalysing, doing and reviewing*. Catalysing change had been seen as a key component of MHL; it was achieved by bringing key members of two very different teams together to discuss the common problem of improving care for LTMI. This simple strategy of face to face contact was valued regardless of the sophistication of the practice, and in most cases resulted in placement of a link worker and some kind of 'joint work'. However, there were reports from both primary care and mental health that initial enthusiasm was easily tempered if reservations from the other team were detected.

Having the facilitator supporting practices in *doing *the work, by providing limited knowledgeable manpower was productive when assisting poorly developed practices to develop basic registers and recall systems, but ineffective when focused on developing more sophisticated in-house electronic systems of care. The importance of the *review *function of the intervention was highlighted by a series of negative cases of intervention practices which had failed to progress with service development in the form of either productive liaison or systems development: the facilitators had either failed to recognise the lack of progress or were unable to help provide a solution. While a *review *function had been incorporated into the original facilitation model, it had not been sufficiently emphasised in the training or implemented in the field.

### Integrating results of the Realistic Evaluation with those of the RCT

Table 3 represents the results of both components of the evaluation for each outcome level. The service development questionnaire used in the RCT reported significant improvements in practice systems for registers, reviews, and the liaison function (mean 2.9 vs 0.7, Mann Witney, p = 0.003). The realistic evaluation provided a more in-depth examination revealing weaknesses in the extent of systems development and the endurance of the liaison function. The intervention was effective in stimulating plans for development and bringing both parties together. In several practices both parties attested to productive liaison relationships. The intervention failed at times to ensure initial success and review progress. Improved GP satisfaction with liaison found in the RCT (adjusted difference of 0.46, CI -0.74 -0.18, p = 0.001) accord well with the stories of improved liaison and resultant care improvement within the Realistic Evaluation. The audit of records in the RCT demonstrated no difference in primary care based mental health or physical review. This is supported by the reports of the interviewees, with no practices setting up sustained proactive recall systems.

**Table 3:** Comparison of different outcomes between the RCT and the process evaluation

**Type of outcome examined**	**RCT**	**Process Evaluation**
Facilitated intervention	()	++/-

Practice systems	++	+/0

Liaison	++	++/0

GP satisfaction	+	+/-

Patient satisfaction/unmet need	0	()

Process of care	0	+/0

Mental Health and relapse	+	()

() = no information gathered;

0 = no difference found between control and intervention;

+ = positive findings in favour of the intervention;

- = negative findings against intervention;

/= mixed findings.

Overall, the results of both evaluations suggest that liaison based on case based discussions, often about patients in crisis, resulted in improved mental health care at the interface in a significant minority of practices. This may have been sufficient to be the cause of the reduced relapse rates seen in intervention practices in the randomised controlled trial.

## Discussion

This is the first known use of Realistic Evaluation as an accompanying process evaluation for an RCT. It provides insights into the interaction between components of the intervention and the external context in which it operates. Themes related to the strengths and weaknesses of the underlying mechanisms of the facilitation emerged with respect to *catalysing*, *doing *and *reviewing*. These help explain both how the intervention operated and the mixed results of the trial.

### Critique of the intervention

Facilitation as the basis for bringing about health services development, has been used for some years, but its effectiveness and internal processes are not well understood [23]. Catalysing change was evident across the outcome groups. By bringing people together, eliciting and suggesting ideas for joint working and encouraging plans for appointment of link workers, regular meetings and involvement in other joint work, as well as systems development, Mental Health Link had a significant impact. Positive impact and joint work was most often in the form of productive relationships between link workers and their host PHCT, with joint discussion of clinical cases and problem solving. With hindsight liaison about clinical cases might appear to have been obvious as the focus for link worker activity, but it was originally conceived as one of many roles [24]. On occasions catalysing change resulted in the negative experience of unproductive joint work. These negative outcomes may have resulted from a failure of the intervention to facilitate optimal decisions based on a problem analysis.

The 'doing' function of the intervention was present in the development of systems for chronic disease management. Facilitators worked alongside members of the PHCT to construct registers and databases. They provided expertise or manpower. This 'doing' function reflects a tension inherent in facilitation: whether to empower by supporting the development of individuals and relationships within teams [25] or by boosting teams with additional skills or resources [26].

The 'reviewing' function was probably the Achilles' heel of the intervention: performance was not adequately monitored and analysis of setbacks and problems insufficient. Reviewing is also little mentioned in most guides to facilitation, and is a component which is likely to be required when attempting to bring about change in the public sector where incentives for rapid development are often lacking. Negative contextual factors interacted with weaknesses of the intervention: reluctance to engage in collaborative activities; inflexibility of IT systems; and competing priorities.

There was also a weakness in the model of chronic disease management: there was insufficient emphasis, outlined in the original conceptual framework [17], for ensuring that PHCTs and community mental health teams focused on pro-actively reviewing patients as a means of detecting unmet need, either through joint clinical case discussions or by inviting patients in for a face to face review. The development of registers and templates rather than actually reviewing care, all too easily became a focus for activity. This mirrors findings in the Liverpool study where practice mental health registers were set up but often not used [27]. Recent changes to the GP contract in the UK [28], with incentives for recalling patients with schizophrenia and bipolar disorder for review of their physical and mental health care, have driven the development of simple systems of recall and review in most practices. Proactive physical care has probably improved but co-ordination of mental health care is still problematic with little liaison between primary and specialist services. Practice based commissioning and payment by results might provide the incentives to redesign care at the interface, and set up the kind of low intensity shared care described by the two practice based community psychiatric nurses in our case studies.

### Methodological issues

The methodological limitations include the use of retrospective interviews, lack of a user voice, potential bias due to the interviews being carried out by RB, the single setting in south-east London, and the untried use of Realistic Evaluation in conjunction with a health services research trial [20]. Retrospective interviews have disadvantages due to selective recall, but prevent the interview process from becoming a potent force for change during the trial period. The method could be criticised for not incorporating the views of those with mental health problems; however we saw the practitioners as the people experiencing this organisational intervention, and those with long term mental illness were unlikely to have been very aware of it. The potential for bias caused by RB carrying out the interviews was recognised early and interview questions emphasised the place for robust critique.

Realistic Evaluation provided a useful framework for ensuring the data capture and facilitating analysis of the causative potential of both context and intervening mechanisms. Observations about the utility of the Realistic Evaluation approach include: at times it is difficult to define whether something is a context or mechanism when constructing CMO configurations; an outcome can become a context or a mechanism in a subsequent event; CMOs at the psychological level can exist within CMOs relating to organisational issues. Our data demonstrated the importance of feedback loops, for example where early positive experiences encourage other positive experiences and increase the chance of a link worker becoming accepted within a primary care team; Realistic Evaluation does not normally incorporate such feed back loops. In summary, Realistic Evaluation is a useful tool but the principles should be flexibly not slavishly adhered to.

### Evaluation of complex interventions

We propose two adaptations of the model in which interventions evaluated in randomised controlled trials can be "out of control" [5]. Firstly, there is a difference between key functions designed to bring about change (only present in the intervention) and those functions which are generic systems of service provision (which may well develop in control and intervention sites in response to local or wider policy initiatives). While interconnected, it is important to consider them separately.

**Figure 2:**
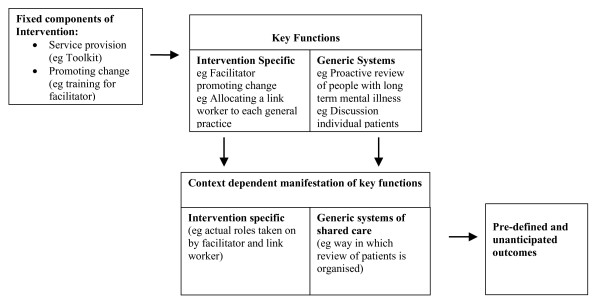
**Conceptual relationship between fixed components, key functions, context dependant manifestation and outcomes of complex interventions**.

Secondly, both these types of key functions may manifest themselves differently in different contexts. There are some components of the intervention, which are fixed in form, and other components which are flexible. In this trial we had a core fixed reproducible intervention: training of facilitators, a toolkit and payment to GP practices. This was accompanied by explicitly flexible components of the intervention, designed *a priori*, to develop the generic components or systems of shared care required to deliver pre-specified improvements to health and care. Keeping with Hawe et al's nomenclature, several key or essential functions *were *revealed in the study: Mental Health Link's change mechanism (catalysing, doing, reviewing); and the resulting shared care (liaison, case discussion, review and recall). While the study highlighted which of the *a priori *functions were critical, these took on *different forms *in different contexts as depicted in Figure 2[5]. Different team make-ups, personalities and economic and political conditions meant that different types of systems of review and liaison developed.

The multiple case study approach, utilising the Realistic Evaluation framework, first documented what had occurred (the different forms) as a result of the interaction of the intervention with the context as individual case studies, and then examined across cases the mechanisms underlying the intervention, revealing the critical functions: those which emerged as being universal and important in any context. Some of these, such as the catalyst function of holding joint meetings and placing link workers, were in the original design. The importance of link workers discussing individual cases with GPs, had not been seen as the critical component of liaison, but was given primacy in the revised model.

### Implications

Future evaluations of complex health promotion or health and social care interventions could incorporate the approach taken in this study as a way of monitoring and understanding interactions with the external context and within the 'black box' of the intervention process. A number of arrangements are feasible. During exploratory (Phase II) trials a prospective mixed qualitative and quantitative enquiry can identify the key functions, of both the change mechanisms and generic service changes, represented by the diverse forms in the field. Such an ongoing process evaluation will inevitably have an impact on the intervention as it proceeds; enquiry about cause and effect based on Realistic Evaluation principles can also be incorporated into the intervention in order to strengthen its internal evaluative management function. In those interventions proceeding to a fully powered cluster randomised controlled trial (Phase III), the process evaluation should be operationally split from the intervention with the collection of prospective quantitative data and retrospective qualitative enquiry conducted by the research team [11].

## Conclusion

The core functions of this complex intervention can be divided into those specific to the change intervention (facilitation) and those related to the specific services (chronic disease management for LTMI). Catalysing change contributed to a range of outcomes, however the reviewing function was, in many instances, insufficient to help overcome problems or ensure sustainability. This process evaluation has emphasised the requirement for both improved liaison and the development of systems for review as core functions of chronic disease management.

The RCT showed fewer patient relapses in the intervention arm, which the process evaluation has suggested may have been due to sporadic but intense improvements in the liaison function rather than systems of recall. The addition of a Realistic Evaluation based process evaluation has provided information to interpret the results of the RCT and has also provided insights into the core functions of the intervention.

## Competing interests

RB led the development of the Mental Health Link intervention.

## Authors' contributions

RB conceived the study, led on design, data collection and analysis and drafted the manuscript. IN and SR participated in the design and analysis of the study and contributed to writing the manuscript. RJ participated in the design of the study and contributed to writing the manuscript. All authors read and approved the final manuscript.

## Pre-publication history

The pre-publication history for this paper can be accessed here:


